# Fake News and social Cognition During The SARS-COV-2 Pandemic: Initial Approach Towards understanding Belief In Misinformation

**DOI:** 10.1192/j.eurpsy.2022.2264

**Published:** 2022-09-01

**Authors:** M. Cordoba-Delgado, J. Molina-Paredes

**Affiliations:** Pontifical Xavierian University, Psychiatry, Bogota, Colombia

**Keywords:** Covid-19, Fake News, Politics, social cognition

## Abstract

**Introduction:**

Infodemic is a new term which refers to rapidly spreading information from both reliable and unreliable sources in the form of news and publications regarding the COVID-19 pandemic, which requires proper management strategies on its own to prevent the spread of fake news. This is especially relevant in a global state of alert where the fear of contagion is a common denominator and is reflected upon people’s behaviors within a crisis context. Van Bavel et al (2020) affirm “Emerging research is using social science to understand and counter the spread of fake news”, and furtherly emphasize on the limitations of Fact Checking as the main approach to hinder such spread

**Objectives:**

Test the association between sociomoral cognition, religiousness and political identity, and belief in COVID-19 Fake News.

**Methods:**

Online-based survey applied through opportunity sampling. Demographic variables political and religious orientation, RMET and B-IRI, and two dimensional utilitarian dilemmas were used and independent variables, and a selection of true and fake news in order to measure participants’ belief in the latter as a dependent variable.

**Results:**

Morality (R2 = 0.08, p < 0.001), social cognition (R2 = 0.05, p < 0.05), and political and religious orientation (R2 = 0.1, p < 0.000001) predicted belief in COVID-19 fake news. On the other hand, no variables were found to predict belief in fake news unrelated to the pandemic.

**Conclusions:**

Higher impartial beneficence and more years of formal education point toward an evidence-based reasoning, while religiousness and affinity with right-wing ideals has been associated with intuition-based reasoning, thus affecting judgement accuracy.

**Disclosure:**

No significant relationships.

